# Lost in translation? Conceptions of privacy and independence in the technical development of AI-based AAL

**DOI:** 10.1007/s11019-022-10126-8

**Published:** 2022-11-08

**Authors:** Kris Vera Hartmann, Nadia Primc, Giovanni Rubeis

**Affiliations:** 1 Institute for History and Ethics of Medicine, Faculty of Medicine, Ruprecht-Karls-Universität Heidelberg, Heidelberg, Germany; 2 Department of General Health Studies, Division Biomedical and Public Health Ethics, Karl Landsteiner Private University for Health Sciences, Krems, Austria

**Keywords:** Privacy, Self-determination, Ethics, Ambient assisted living, Artificial intelligence

## Abstract

AAL encompasses smart home technologies that are installed in the personal living environment in order to support older, disabled, as well as chronically ill people with the goal of delaying or reducing their need for nursing care in a care facility. Artificial intelligence (AI) is seen as an important tool for assisting the target group in their daily lives. A literature search and qualitative content analysis of 255 articles from computer science and engineering was conducted to explore the usage of ethical concepts. From an ethical point of view, the concept of independence and self-determination on the one hand and the possible loss of privacy on the other hand are widely discussed in the context of AAL. These concepts are adopted by the technical discourse in the sense that independence, self-determination and privacy are recognized as important values. Nevertheless, our research shows that these concepts have different usages and meanings in the ethical and the technical discourses. In the paper, we aim to map the different meanings of independence, self-determination and privacy as they can be found in the context of technological research on AI-based AAL systems. It investigates the interpretation of these ethical and social concepts which technicians try to build into AAL systems. In a second step, these interpretations are contextualized with concepts from the ethical discourse on AI-based assistive technologies.

## Introduction

Ambient assisted living (AAL) technologies combine technologies such as sensor-networks and monitoring systems with telehealth technologies, wearable sensors, and sometimes also assistive robotics (Manzeschke et al. 2016; Offermann-van Heek et al. 2019; Sapci and Sapci 2019). The latest generation of AAL is based on Artificial Intelligence (AI), i.e. methods of machine learning and Big Data applications. These systems allow to collect and process large amounts of user data. Based on this data, care services can be tailored to the specific needs and resources of the individual user. The aim is to enable older adults to remain in their home environment for as long as possible and live a mostly independent life.

Although AAL yields many benefits, ethical concerns have been raised. One major ethical issue is the supposed independence of users. In AAL research, independence is widely understood as the ability to cope with everyday life without the help of third parties, especially informal and professional nursing care. As mentioned above, this is one of the main goals of AAL. It has been argued that the use of AI to monitor and regulate user behavior may lead to a standardization of lifestyle (Manzeschke et al. 2016). This would severely undermine user autonomy or self-determination, another aspect that is believed to be of great importance in the context of assistive technologies. Autonomy and self-determination are often used as synonyms in the context of AAL. In a very general sense they designate the ability to live one’s life and make one’s own decisions according to one’s own values and rules, without being restricted by anyone else. Technology-induced standardization of everyday life could therefore entail a loss of self-determination in order to be able to live on your own in your own home. Another ethical concern is the threat to privacy. The collection and processing of large amounts of sensitive user data raises the questions how this data is protected and who has access to it (Pirzada et al. 2021). Furthermore, the use of surveillance and monitoring technologies in the home environment is seen as a massive intrusion into privacy, blurring the borders between the medical and the private and thus leading to a medicalization of the home environment (Mortenson et al. 2015). As will be discussed below, privacy should also be understood as closely related to self-determination. The latter encompasses the right and ability to decide who has access to one’s sensitive personal data.

Given these ethical concerns, the concepts of independence, self-determination as well as privacy require a thorough examination in AI-based AAL. Since AAL research is an interdisciplinary field, combining research from medicine, gerontology, nursing science, engineering and computer science, an ethical investigation should focus on how these concepts are used in these different disciplines. This could give us an understanding of the underlying narrative that affects not only research, but also technology design.

The aim of this paper is to make a first step in that direction by analyzing how the concepts of independence, self-determination as well as privacy are used in the technical disciplines involved in research on AI-based AAL, i.e. engineering science and informational science. In order to do so, we have conducted an encompassing qualitative analysis of the literature on AI-based AAL in these disciplines, focusing on independence, self-determination and privacy. The paper presents the results of this analysis and contextualizes them with the ethical discourse. In a final step, we will shortly outline several aspects that may contribute to enrich these concepts for a more appropriate use in the technical disciplines.

## Method

In order to relate the use of the concepts of independence, self-determination and privacy in engineering and computer science to the ethical discussion of these concepts, we conducted a qualitative content analysis (Mayring 2000) of scientific articles from the fields of engineering as well as computer and information science. To obtain the material for the analysis, we conducted a literature search in online search engines. After comparing different search engines for scientific articles, we decided to use the search engine “PubMed”, which is focused on biomedicine and life science. Additionally, we used “ScienceDirect”, as this search engine is specialized in science and technology as well as medicine, health and life science. As we were interested in the special field of Ambient Assisted Living, the search terms were limited to “Ambient Assisted Living” and “AAL” (see the limitations regarding the search terms in the section “limitations”). In the two search engines a wide range of articles suitable for our research were listed with only a small overlap. The inclusion criteria were English language and research articles from scientific journals only (no monographs, anthologies or dissertations). Table 1 shows the search terms as well as the process of compiling the research sample. As we were interested in the usage of ethical concepts by computer and engineering scientists, the articles examined are exclusively articles in which a developed technology is presented and no or very little interdisciplinary cooperation is visible. The developed technologies are listed in Table 2. The sample contains papers on research and development regarding Human activity recognition (HAR), sensor technologies, algorithms or even complete AAL systems among other technologies as shown in Table 2.

**Table 1:** Compilation of the research sample

Compilation of the research sample	
1. PubMed and science-direct search, keyword „AAL“	2.221
2. PubMed and science-direct search, keyword „ambient assisted living“	2.217
3. Title and abstract scanning; exclusion of thematically not relevant papers, duplicates and other publication forms	− 3.443
4. Exclusion of not accessible articles via German university libraries	− 90
5. Exclusion of reviews & surveys (PubMed only)	− 87
6. Exclusion of other disciplines than engineering and information and computer science (PubMed only)	− 168
7. Lexical search in papers for the use of ai-techniques (search terms: “artificial intelligence”, “machine learning”, “neural network”, “deep learning”)	− 395
Number of articles in research sample	**= **255

**Table 2:** Technical focus (summarized)

Technical focus (summarized)	Articles (n = 255)
Activity recognition and monitoring, incl. event, gait & posture recognition	93
AAL-Systems (incl. frameworks, middleware), ambient intelligence, smart home, pervasive systems, internet of things	51
Artificial intelligence and special AI-methods	25
Sensor Networks	20
Health related systems	17
Fall detection & prediction	13
Robotics	11
Emotion recognition	5
Special interfaces	5
Other (simulation, technical evaluation, etc.)	15

In the next step, we conducted a qualitative content analysis of the articles. The analysis was performed by one of the authors (KVH, sociologist). Firstly, the ethically relevant passages were identified in the articles. Secondly, a lexical search was conducted with the analysis software MaxQDA to identify text passages in which the relevant topics were addressed. In a third step, a qualitative analysis of the relevant passages was conducted and the concrete meaning of the concepts in question (self-determination and privacy) was elaborated inductively. In a last step, the results of the inductive analysis were discussed in our research team (KVH, sociologist, NP, GR, both philosophers/ethicists) to elaborate the ethical-normative implications that the identified concepts have against the background of the concepts of “activities of daily livings”, active ageing, as well as the different ethical dimensions of privacy (see section “Ethical analysis”). In the next chapter, the key findings of our analysis will be presented in form of a narrative synthesis.

## Results

### Independent living and self-determination as key goals of AAL

The social relevance of the research topic or the developed technology is mainly mentioned in the introductions of the papers. The demographic shift, i.e. the increasing number of people older than 65 years in the near future and the associated, increasing and unmet need for professional care is emphasized in the majority of papers (e.g. Liciotti et al. 2020; Sarabia-Jácome et al. 2020; Xu et al.  2020). On a socio-political level, technology is expected to compensate the increasing unmet need by enabling older people to live a self-determined, independent life without the help of others for as long as possible. Thus, the concepts of self-determination and independence are seen as crucial goals of AI-based AAL technologies: Technology is supposed to enable older, disabled, and/or chronically ill people to live independently from the help of others. The high significance of independence manifests in the fact that it is explicitly named as crucial goal of AAL in 134 papers (e.g. Fahad and Tahir 2020; Guerra et al. 2020; Tian and Zhang 2020). Independence tends to be used as a surrogate or even a synonym for autonomy and/or self-determination. Typical sentences in these contexts are e.g.: “[The technology] may help in preserving quality of life of older adults, allowing them to maintain their independence and live longer and safer in their own home” (Mora et al. 2018, p.13), “[it] contributes to elderly independent and more autonomous living” (Bleda et al. 2019, p. 12), or “[it] can promote the independent living of elderly in a safe and comfortable environment of their own homes, for an extended period of time.” (Fahad and Tahir 2020, p. 1).

Independence is often linked to the activities of daily living (ADL), a concept derived from nursing science (see e.g. Shelkey and Wallace 1999), which plays a central role especially in HAR. This concept is used in intelligent pattern recognition to detect deviations in the users’ behavior that may be signs of illness, functional deterioration, or dangerous situations (e.g. falls) and may require external intervention (e.g. Diraco et al. 2017, 2019; Eisa and Moreira 2017; Gochoo et al. 2019). With the detection of previously defined activities, an indirect monitoring should take place: Based on the different sensor data, the general state of the user will be measured and evaluated. However, the focus in activity recognition is not a detection of the entire human activities, but on activities that are considered important for the health status, which is why mainly bodily activities like moving, eating, drinking, toilet usage and personal hygiene are operationalized. Variations of ADL-concepts are mentioned in 136 papers of the research sample (e.g. Uddin et al. 2020; Van Woensel et al. 2020; Vourganas et al. 2020).

**Fig. 1:**
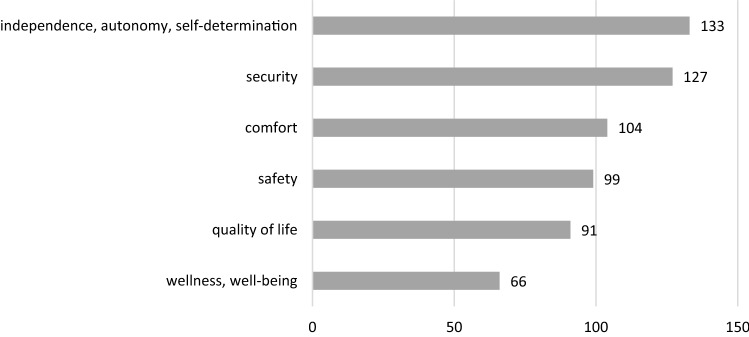
Frequencies of papers with ethical terms

Empowering older adults to live an independent life is often not defined as an end itself, but as a crucial factor of an improved quality of life (e.g. Calderita et al. 2020; Karakostas et al. 2020; Martín et al. 2020), well-being, or wellness of older adults (e.g. Grgurić et al. 2019; Guerra et al. 2020; Jovanov 2019). Key factors linked to this improvement are providing more comfort (e.g. Stojanova et al. 2019; Calderita et al. 2020; Golestan 2020) in the home environment as well as security (e.g. Al Machot et al. 2019; Leonidis et al. 2019; Helal and Bull 2019) and safety (e.g. Guerra et al. 2020; Tian and Zhang 2020; Vourganas et al. 2020). Figure 1 shows the frequencies of papers using the aforementioned terms.

### Variations of privacy

Privacy is one of the central ethical concepts addressed in the context of AAL (Guerra et al. 2020; Tian and Zhang 2020; Vourganas et al. 2020). Five articles describe a tension between privacy and the functionality of AAL systems (Aquino-Santos et al. 2013; Lampoltshammer et al. 2014; Leitner et al. 2014; Padilla-López et al. 2015; Eisa and Moreira 2017): This tension arises from the fact that in order for AAL-systems to work properly, an intrusion into privacy is necessary. However, the term privacy is used very differently in the research sample. The complexity of the concept of privacy in all its dimensions often leads to being regarded as something merely subjective: For example, users have the “perception” or “feeling of reduced privacy” (Leitner et al. 2014, p. 13,499) and the so-called privacy issues are described as an ethical problem that has to be considered in technology. In the following, the different dimensions of privacy as discussed in the analyzed papers will be explored.

**Fig. 2:**
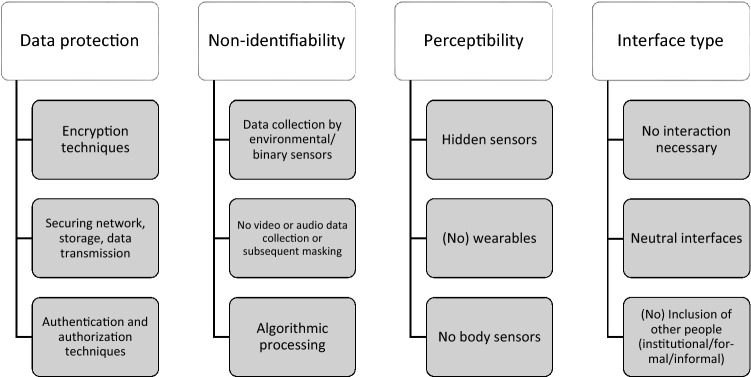
Variations of privacy

Figure 2 shows a compilation of the variations of privacy in the sample, which are discussed in the next paragraphs.

#### Data protection

The first variation of privacy discussed in the articles is the issue of data protection and its technical implementations (e.g. Guo et al. 2020; Jayaram and Prabakaran 2020; Vourganas et al. 2020). In contrast to the mere mention of privacy issues, various technologies relating to data protection are described and/or put to use here. These range from securing data storage, data transmission, data processing and network as well as the management of access to encryption techniques and access restrictions (authorization and authentication).

A typical framing is that the developed technology “preserves privacy“ (Jayaram and Prabakaran 2020, p. 1) of the users themselves or their data (Alsina-Pagès et al. 2017, p. 17). Guo et al. (2020) refer to a "threat of privacy leakage" (2020, 407). This example illustrates a metaphorical concept of privacy, which runs (as a data stream) through a pipe, can therefore also “leak out” and must be protected by the data protection technologies.

#### Non-identifiability

Non-identifiability is a borderline case between data protection and local privacy protection. It can be differentiated into three subcategories: First, there are techniques that anonymize data in the process of data collection (e.g. Ghayvat et al. 2019; Guerra et al. 2020; Xu et al. 2020). Second, anonymity can be ensured in the information processing (Armentia et al. 2015) or postprocessing (e.g. Leitner et al. 2014; Padilla-López et al. 2015; Navarro et al. 2018). Third, some authors state that the use of cameras (e.g. Sarabia-Jácome et al. 2020; Tian and Zhang 2020; Vourganas et al., 2020) and microphones (Olaru and Florea 2014; Tunca et al. 2014; Navarro et al. 2018) in particular should be excluded, as these types of sensors reveal too much information about the user. The following technologies are considered to be particularly anonymity-preserving: (a limited number of) environmental sensors, binary sensors like pressure, motion or electricity (Garcia-Ceja 2018), accelerometers (acceleration sensors, e.g. in smartphones), radio-frequency identification (RFID) sensors, infrared, sound, heat, or blind/computer vision and depth cameras (e.g. Gingras et al. 2020; Sarabia-Jácome et al. 2020; Xu et al. 2020). By using these sensors, anonymization already takes place in data collection. The project by Guerra et al. (2020) can be cited as an example here: Images of users are not captured, but their skeletons and movements are “estimated” with algorithms, and the technology thus works “intrinsically anonymous” (Guerra et al. 2020, 2). Somewhat simpler is “negative monitoring”: in order not to cover all living areas of the user, algorithms can be used to infer positive values from negative ones. An example is the project by Olaru and Florea (2014), where no sensors are installed in the bathroom for privacy reasons, but the system can infer that the user is currently using the bathroom (2014, 11,127). However, a variant of anonymization can also be the subsequent anonymization of camera recordings, such as in the project by Padilla-Lopez et al. (2015), in which the images are covered with blurred silhouettes in post-processing. An overview of so-called “visual privacy” measures can also be found there. Non-identifiability can be classed with informational as well as local privacy as its about data security, data protection (from unwanted access) as well as protection of the personal refuge and thus also the body of the user from unwanted observation.

The extensibility of the term privacy can also go so far as to limit privacy to non-identifiability. Rus et al. (2018), for example, developed a couch that is able to recognize emotions. Emotion recognition has so far been done mainly by analyzing images, but the authors see an advantage in using sensors in the couch: “[…] in our increasingly observed society, more privacy-aware methods are worth exploring that do not require facial images, but instead look at other physiological indicators of emotion. In this work we present the Emotive Couch, a sensor-augmented piece of smart furniture that detects proximity and motion of the human body.“ (Rus et al. 2018, p. 263) Observation or surveillance here is only seen as a visual process and not in its whole sense or all its different forms. Emotion recognition with sensors in a couch is also an invasion of privacy just like the visual detection of faces. Even more: emotion recognition in itself is already an invasion of privacy, and it can be argued that a technology that hides itself from the user is not more privacy aware than one where the surveillance is openly visible.

#### Perceptibility

Another variation of privacy is the perceptibility of the system. This variation of privacy shows itself in the use of the terms unobtrusive, non-intrusive, and non-invasive. Although these terms certainly differ in their meaning, they are widely used as synonyms in the selected papers. These terms and corresponding technologies can be found in 132 articles of the study sample. In addition to the synonymous use of the terms, a very unclear application to the concrete techniques is also noticeable: for example, the use as well as the non-use of wearables is described as unobtrusive (see e.g. Garcia-Ceja and Brena 2016; Grgurić et al. 2019). What these different terms have in common is that they consider the person, their home, and their everyday life as something to be protected from the AAL-technology. In conclusion, the terms indicate a latent reference to a private sphere, which needs to be protected. For example, the technological systems must not disturb the “the people’s environment but making easy their daily activities” (Rodríguez et al. 2015, p. 43) or must not “interfere” with the activities and the “traditional life” (Li et al. 2020, p. 108) of the users. The low intrusiveness is also seen as a possibility for an “easier adaption” by the users and their acceptability (Olaru and Florea 2014, p. 14).

#### Type of interface

Another variation of privacy can be found in limited interaction requirements with the system (Mollaret et al. 2016; Li et al. 2020; Sarabia-Jácome et al. 2020), which is also often described as non-intrusiveness or unobtrusiveness This includes hiding technical processes as much as possible so as not to overwhelm people who lack technical expertise. Ghidoni et al. (2014, 1317) refer to this requirement as allowing the system to work with “non-cooperating” people. Furthermore, the dimension of interface includes the way in which interventions take place in case of (assumed) emergencies or deteriorations in health status: Does the system intervene by notifying formal or informal caregivers (e.g. Blasco et al. 2014; Ghayvat et al. 2019) or is there a direct “address” to the user (e.g. Ayari et al. 2016; Mollaret et al. 2016)?

The variety and extensivity of the interpretations of privacy becomes particularly clear when we take a closer look at the description of assistive robots in the sample. Robots that at first glance do not fit the category of hidden technology are also referred to as non-intrusive (or similar) in the sample. An example is the ‘’ubiquitous robot’’ by Ayari et al. (2016), which gives “non-intrusive advices and reminders” (Ayari et al. 2016, p. 18) for a healthy lifestyle. Mollaret et al. (2016), on the other hand, describe the non-intrusiveness of their robot as starting an interaction “only when it detects the user’s intention to do so, and before that the robot monitors the person with an RGB-D camera and audio sensor.“ (Mollaret et al. 2016, p. 80).

## Ethical analysis

### Independence and self-determination

As we have seen independent living and self-determination are defined as crucial goals of AAL. Although from an ethical point of view these concepts may be regarded as closely connected, they nevertheless have to be clearly differentiated. On the one hand, a person can be dependent on the support of a third party in everyday activities and still lead a self-determined life. On the other hand, as the issue of technology-induced standardization of everyday behavior shows, a person can be independent from the support of a third party and still not be able to lead a self-determined life, as he or she has to stick to the routines that have been identified by surveillance technologies in order not to show any abnormal activity patterns that may trigger system notifications to emergency contacts. Furthermore, a loss of self-determination can also be linked to a loss of privacy, if one is forced to accept that very intimate data are tracked on a daily basis in order to be allowed to live independently in one’s own home. Here too the loss of self-determination is irrespective of the independence from the support of a third party.

In some of the analyzed papers, these concepts are used synonymously, mostly lacking clear definition. Where these concepts are defined, we found a limited meaning. Independence is mostly understood as the absence of the need to rely on the help of other people, which itself is mainly focused on bodily care and housekeeping. Two aspects are crucial in this regard, the focus on bodily care and the ideas of a good life.

The ability to care for oneself on a bodily level stands for material prerequisites that are understood as necessary for living an independent life. This concept of independence is used in engineering and computer science as a surrogate for self-determination and is closely related to the aforementioned “activities of daily living” (ADL). The concept of ADL was initially developed in a clinical setting in the 1960s (Katz et al. 1963; Lawton and Brody 1969) and has since undergone several modifications. In their original paper Katz et al. (1963) assumed that their index can be helpful in practice for medicine, care and rehabilitation, and nursing education, and that deteriorations as well as improvements in the general state of health can be determined with the help of the index. The authors view independence as important for sustaining physical, emotional and social strength (Katz et al. 1963). The Lawton and Brody (1969) index of “instrumental” ADL is also interested in an objective determination of the degree of “functioning” of an individual and the related professional treatment planning. However, ADL are themselves based on normative conceptions of a good life, which needs to be critically reflected from an ethical point of view (Porter 1995). The attractiveness of the ADL-concept in the analyzed papers lies in its potential to measure and operationalize “normal” and “healthy” human behavior. This is why ADL are important components of the technological operationalization of independence in engineering and computer science. One problematic aspect here is the focus on the pathological. The intended measurement of activities by various sensor technologies tends to standardize behavior in terms of a pathologization if they are not highly customizable. That means that the focus on particular ADL and more general on bodily aspects of daily life imply the need for a technology that focuses on the deficits of older adults. The result is a view of older adults as frail and vulnerable and in need of help. This so-called age script (Neven 2015) is written into the technology, determining its use and purpose. With their focus on bodily care, they ignore social determinants of vulnerability, such as ethnicity, gender, or socioeconomic status (Fang et al. 2018). Age scripts thus ignore user diversity as well as the individual needs and resources of older adults by defining them as one homogenous group (Ayalon and Tesch-Römer 2018). The focus on bodily care and specific ADL as a surrogate for self-determination may thus undermine self-determination instead of enabling it.

The second abovementioned aspect concerns the implicit ideas of good life that are written into AAL-technologies. Independence and self-determination are directly linked to an increased quality of life. This implicit assumption is mostly derived from the concept of active ageing, introduced by the World Health Organisation (WHO) (Kuziemsky et al. 2019). According to the WHO, active ageing aims at facilitating health, security, and social participation for older adults in order to enable them to lead an active and largely independent lifestyle. The overarching goal is to improve and maintain older adult’s quality of life (WHO 2002). At first glance, the active ageing-approach seems favorable since it goes beyond the notion of older adults as frail and in need of help. However, this concept rests on certain assumptions that are seldom questioned (Stephen Katz and Calasanti 2015; Pfaller and Schweda 2019). In philosophical terms, concepts of a good life are used with an implicit understanding. Two problems arise here. First, active ageing suggests that it encompasses objectives that are universally desirable. However, living alone in a medicalized and technologically enhanced environment may not be desirable for all older adults (Neven 2015). Second, the assumption that technology use in itself is the right way to realize such a concept of a good life ignores the social determinants of health, which are fundamental factors of the setting in which technology is used. Technology that is designed with an awareness for health disparities and without the explicit goal of facilitating health equity will be useless in any other than an ideal setting. A good life for older adults in terms of agency and social participation can only be achieved through health equity. Being able to lead an active, independent, and meaningful life requires the fundamental ability to participate in society and make use of one’s personal resources. The scope and quality of participation as well as agency depend strongly on the social determinants of health.

### Privacy

Our research shows an extensive and diverse use of the concept of privacy which should hence be contextualized by relating it to an equally encompassing ethical concept. A broad and integrating concept of privacy has been developed by Rössler (2004). Drawing on various philosophical and sociological approaches, she divides privacy – which in her understanding is always relational and crucial for living an autonomous life—into three dimensions: *local*, *informational*, and *decisional privacy*. We can look at these dimensions separately and classify the previously described variations accordingly. On the one hand, privacy is understood as data protection and/or data security, which is closely related to the concept of security and is intended to avert threats from outside (e.g., misuse of data by third parties). This interpretation of privacy can be referred to as *informational privacy*, following Rössler. On the other hand, privacy is also understood by engineering and informational scientists as *local privacy* that is invaded by the system. The conceptualizations of non-identifiability, perceptibility and their technical counterparts can be located in this area. The design of the human-machine interfaces is also part of a privacy conceptualization, which can be understood as part of the *decisional privacy*. In all three conceptions, AI is discussed as an opportunity to protect the privacy of end users.

#### Informational privacy

According to Rössler (2004), informational privacy signifies the control over the personal knowledge that other people (or technical systems) have obtained about ourselves. This implies that we know and to some extent control what others know about us so that we are able to assess our relation to other individuals or institutions. When it comes to AI-based AAL systems, we are dealing with a fundamental trade-off. On the one hand these systems can only function properly if large amounts of personal data are collected and processed. On the other hand, individual health data and other data related to a person’s daily activities and behavior are highly sensitive data that require protection and safety protocols. This issue is mostly tackled by either technical solutions aimed at data safety and security or policies as well as guidelines for privacy protection (Ienca and Villaronga 2019). Both approaches may be paternalistic since they exclude individual decisions about what data to share. Making these decisions implies a certain form of control over one’s own data as well as the technical means for collecting and processing them (Schomakers and Ziefle 2019). That means that the more passive approaches of data security and privacy protection are insufficient to enable informational privacy. Older adults should instead be given possibility to actively opt for or against the sharing and use of their data. One perspective is to apply the concept of granular consent where older adults may choose what data to share with whom for what purpose for every single application instead of giving an overall consent to data use. Another approach is designing modular AAL-systems whereby data sharing-functions may be switched on or off by older adults (Schomakers and Ziefle 2019).

#### Local privacy

Local privacy describes the possibility to retreat in one’s own personal space (Young 2005), where one can withdraw from the views of others, where access is only granted by the inhabitant and where this inhabitant therefore has a chance to try out different approaches of presenting him- or herself to the social surroundings. This refuge therefore is a necessity if we want to control the image others have of us. The space itself and its furniture or scenery can be of importance for this image as well and has thus to be protected from third-party access. This is intended by the concealment of sensors etc. for which the terms “unobtrusive”, non-invasive” and “non-intrusive” are generally used in engineering and computer science (Hensel et al. 2006). However, this form of concealment is highly problematic in several regards. The physical invisibility of certain applications may lead to mental invisibility (Crutzen 2006), which can negatively affect the ability of the user to create his or her self-image. Also, the ability to have a conscious relationship with the AAL-system or his or her surroundings in general may be afflicted. Furthermore, concealing the AI-involvement may also be interpreted as implicit deception (Wangmo et al. 2019). This is especially the case when older adults interact with assistive robots or other conversational agents. Older adults may be led to believe that they interact with a human or at least a human-controlled device when instead they are interacting with an AI-based system.

#### Decisional privacy

The type of the human-machine interface can be classed with decisional privacy. This requires the possibility for the user to interact with the system, give input, or change settings. At the heart of decisional privacy lies the ability to decide about one‘s own life and activities, actions to take or not to take and to decide for oneself if and to whom one wants to be responsive or who one wants to involve in these decisions (Rössler 2004). With the implementation of AI-based AAL, this ability to decide is often undermined. The monitoring and regulation of daily activities, behavior, and in some cases even emotions are crucial elements in this regard. The underlying assumption is that based on individual health data as well as scientific data, objective parameters can be defined for appropriate or desirable behavior. This epistemological approach has normative implications (Morley et al. 2020). There is a certain risk that instead of being objective, these parameters are deliberatively designed in order to enforce a specific behavior, e.g., to save personnel and save costs. This enforcement can take the relatively mild form of nudging (Thaler and Sunstein 2009), i.e. influencing a person’s behavior through subtle actions instead of direct coercion. A person’s behavior is thus influenced in a certain way for the person’s own good, which has been described as benevolent paternalism in the context of AAL (Manzeschke et al. 2016). A more severe form would be disciplining, i.e. enforcing a desirable behavior that serves the interests of others by structuring a person’s environment and scope of action (Hummel and Braun 2020; Rubeis 2020).

Decisional privacy is threatened on several levels. First, the standardization of activities, behaviors, and emotions limits the scope of actions of older adults. AI-based AAL-systems will often interfere as soon as a certain behavior for example varies from the standard, e.g. by informing caregivers to check on the older adult. This can be a frustrating experience that causes the older adult to adopt his or her behavior to said standards. Second, this kind of depersonalized care substitutes human contact by human-machine-interaction without the possibility to opt out. That means that older adults cannot decide against interacting with an AI and chose a human caregiver instead. Third, crucial decisions regarding system design have already been made when the system is implemented. Older adults often have no possibility to decide which data they want to share or which aspects of their daily lives should be accessible to monitoring and surveillance.

## Conclusion

AI-based AAL may offer the opportunity to support older adults in leading a more independent life in their own home environment. Based on the possibility of collecting and processing large amounts of individual data, these technologies may go beyond dealing with illness, impairment, or disability. Combining and integrating data from various sources such as connected sensors or wearables, as well as human-computer-interfaces for telehealth, they provide the opportunity to tailor health services to the individual needs and resources of persons. Instead of ready-made health service that depend on a prefixed notion of what old age means or what older adults need, AI-based technologies might thus be more flexible and easier adaptable to individual needs. Instead of a deficit-oriented focus, AI-based AAL could enable older adults to live an active life and facilitate quality of life through assistive technologies. This could improve the situation of older adults with special health needs in terms of the above-mentioned complex interaction of social determinants.

However, the crucial concepts connected to the goals of AAL, mainly independence, self-determination and privacy, are often used in a superficial and uninformed manner in engineering and informational science. An understanding of the inherent complexity is mostly lacking in these disciplines, which makes it seem like we are dealing with clear-cut and unproblematic concepts. In order for AI-based AAL to realize its full potential and enable an independent lifestyle as well as protect user privacy, enriched concepts of independence, self-determination and privacy as outlined in this paper should be used in these disciplines. The following aspects should be considered:

- Independent living and quality of life cannot be defined by bodily care alone. Individual user characteristics, the needs and resources of persons, should be considered when implementing AI-based AAL. One strategy could be to diversify the training data of the algorithms involved. This could be done by integrating social determinants when defining proxies and biomarkers for training purposed (Walsh et al. 2020). Another approach is to actively engage stakeholders and community in the design process (Fohner 2019). The key is user participation in the design process and feedback loops after the technology has been implanted. This approach allows to tailor AAL-technologies to the specific needs of a certain group or context of use. In addition, age scripts that are used for designing and/or have been implemented have to be critically reflected upon in order to prevent standardization of user preferences.
- Older adults do not form one homogenous group, but mirror the whole range of diversity in society when it comes to gender, ethnicity, and socio-economic status. Defining equity as an explicit goal of technology already in the design process can be a way to acknowledge this diversity and at the same time enable agency as well as social participation. When designed with equity as a main objective and informed by user needs and benefits, AI-based technologies could have the potential to counter the standardization of behavior. Frameworks for building AAL that entail ethical values in this regard are already available, such as The Social Justice Framework for Bridging the Digital Divide, (Fang et al., 2019). By acknowledging that health structures are often built for individuals in more advantageous social positions, this framework allows to address access inequalities at an early stage in the product cycle. Thus, differentiation across groups according to their access opportunities can be built into the technology, thus allowing it to be integrated in an equity-focused healthcare setting.
- Since older adults have different demands due to the different characteristics and biographies, the perceptibility of the systems must be individually negotiated with the user. While some may be more likely to develop anxiety if they do not perceive the monitoring themselves, this could work well for others. As soon as AI-technology or the interaction with AI-based agents is involved, older adults should be informed about this fact. They should also have the opportunity to contact human agents when they do not feel comfortable with interacting with AI-systems.
- Data use and access as well as security standards have to be fully disclosed when collecting and processing large amounts of individual data. What is crucial here is to preserve and enable the ability of users to make autonomous decisions by granting explicit and affirmative consent (Wangmo et al., 2019). Opt-in mechanism, e.g. granular consent should be provided that enable older adults to consent to each purpose of data use individually and to decide with whom data should be shared.

This list is far from being complete. There are still more barriers to implementation, such as privacy policies and data security protocols, digital literacy, and the special needs of different groups of older adults, e.g. people with dementia, that have to be addressed in further research. Our research has shown that there is still translation work to be done in AAL research between computer science and engineering on the one hand and ethics on the other. Furthermore, research is needed that highlights the limitations of AAL systems, e.g., their dangers of social isolation.

## Limitations

The analysis was limited to articles that are linked to the keywords “AAL” and “Ambient Assisted Living”. This excludes articles that use other, related keywords, such as “gerontotech”, “agetech”, “domotics” etc., but which may describe similar individual technologies. With the present study, no conclusions can be drawn about the usage of ethical terms or concepts in these other areas or explicitly interdisciplinary approaches.
